# Correction: Study of chromium, selenium and bromine concentrations in blood serum of patients with parenteral nutrition treatment using total reflection X-ray fluorescence analysis

**DOI:** 10.1371/journal.pone.0249347

**Published:** 2021-03-24

**Authors:** The PLOS ONE Staff

## Notice of republication

An incomplete, earlier version of this article was published in error. The Conclusion section was erroneously omitted at the end of the article. The publisher apologizes for the error. This article was republished on March 8, 2021, to correct for this error. Please download the article again to view the correct version. The originally published, uncorrected article and the republished, corrected article are provided here for reference.

## Supporting information

S1 File Originally published, uncorrected article. (PDF)

S2 File Republished, corrected article. (PDF)
