# Supplementary material for: Study of chromium, selenium and bromine concentrations in blood serum of patients with parenteral nutrition treatment using total reflection X-ray fluorescence analysis
Source: PLoS One. 2020 Dec 15;15(12):e0243492. doi: 10.1371/journal.pone.0243492 (PMC7737982; doi:10.1371/journal.pone.0243492)
Supplement: S1 Fig — In the spectrum Cr, Se and Br K-α lines are observed. The intensity of the line is converted to element concentration. (PDF) [file pone.0243492.s001.pdf]

S1 Fig

SUPPORTING INFORMATION

Article title:
Study of chromium, selenium and bromine concentrations in blood serum of patients with parenteral nutrition treatment using total reflection X-ray fluorescence analysis

Journal name:
PLOS ONE

Author names:
M. Pierzak, A. Kubala-Kukuś, D. Banaś, I. Stabrawa, J. Wudarczyk-Moćko, S. Głuszek
corresponding author: phone/fax: +48 41 349 64 63; e-mail: aldona.kubala-kukus@ujk.edu.pl (AKK).
Affiliation and e-mail address of the corresponding author:
Institute of Physics, Jan Kochanowski University, Uniwersytecka St. 7, 25-406, Kielce, Poland, e-mail: aldona.kubala-kukus@ujk.edu.pl

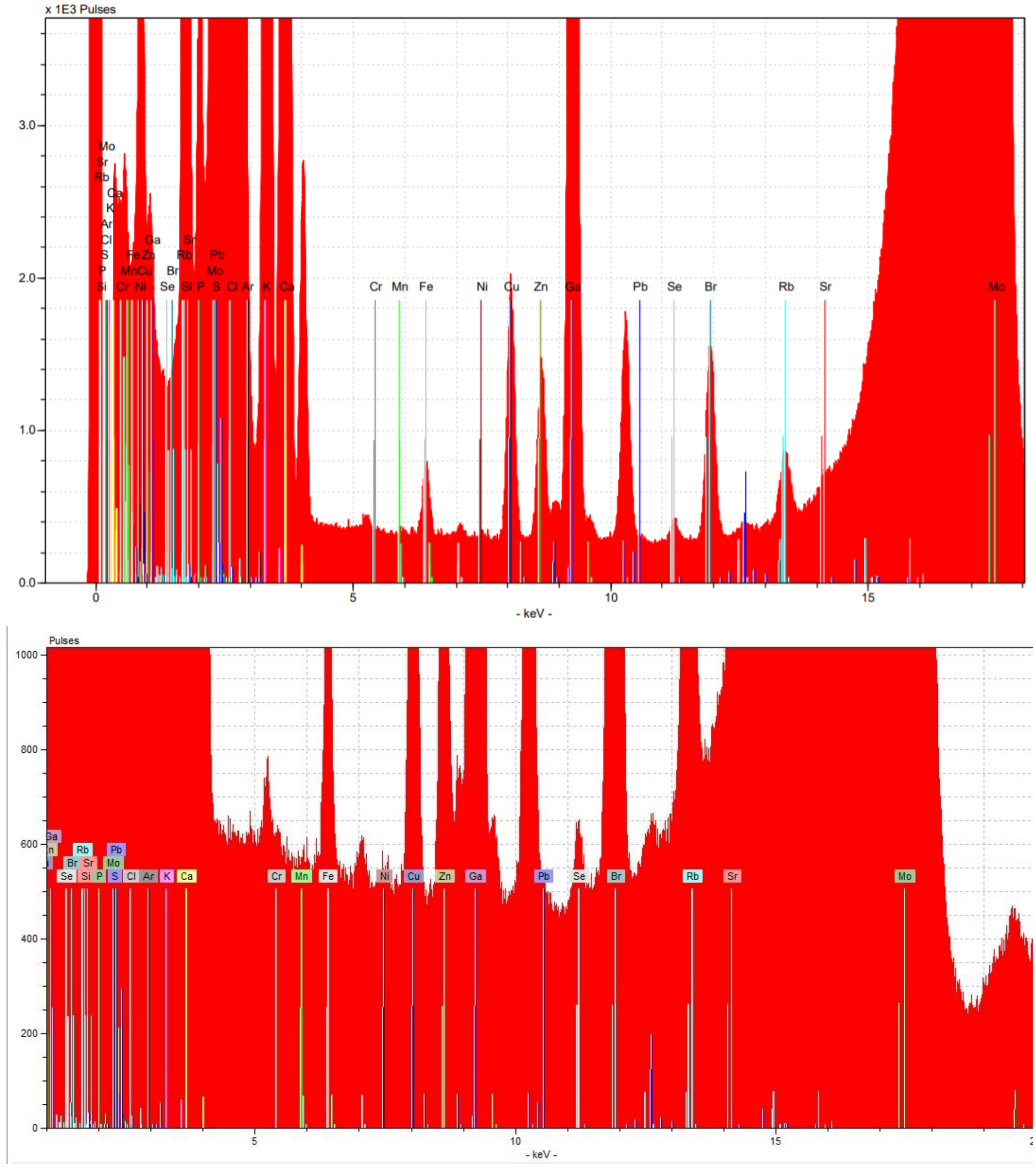

Spectra of the X-ray characteristic radiation emitted from the serum sample, obtained using total reflection X-ray fluorescence analysis (S2 Picofox spectrometer). In the spectrum Cr, Se and Br K-α lines are observed. The intensity of the line is converted to element concentration.
