# Supplementary material for: Correction: Study of chromium, selenium and bromine concentrations in blood serum of patients with parenteral nutrition treatment using total reflection X-ray fluorescence analysis
Source: PLoS One. 2021 Mar 24;16(3):e0249347. doi: 10.1371/journal.pone.0249347 (PMC7990194; doi:10.1371/journal.pone.0249347)
Supplement: S1 File — (PDF) [file pone.0249347.s001.pdf]

PLOS ONE

Check for updates

RESEARCH ARTICLE

Study of chromium, selenium and bromine concentrations in blood serum of patients with parenteral nutrition treatment using total reflection X-ray fluorescence analysis

Monika Pierzak[1], **Aldona Kubala-Kukuś**[2,3]*, **Dariusz Banaś**[2,3], **Ilona Stabrawa**[2,3], **Jolanta Wudarczyk-Moćko**[3], **Stanisław Głuszek**[4,5]

1 Institute of Health Sciences, Jan Kochanowski University, Kielce, Poland, **2** Institute of Physics, Jan Kochanowski University, Kielce, Poland, **3** Holycross Cancer Center, Kielce, Poland, **4** Institute of Medical Sciences, Jan Kochanowski University, Kielce, Poland, **5** Clinic of General, Oncological and Endocrinological Surgery, Regional Hospital, Kielce, Poland

* aldona.kubala-kukus@ujk.edu.pl

OPEN ACCESS

Citation: Pierzak M, Kubala-Kukuś A, Banaś D, Stabrawa I, Wudarczyk-Moćko J, Głuszek S (2020) Study of chromium, selenium and bromine concentrations in blood serum of patients with parenteral nutrition treatment using total reflection X-ray fluorescence analysis. PLoS ONE 15(12): e0243492. https://doi.org/10.1371/journal.pone.0243492

Editor: Oscar Millet, CIC bioGUNE, SPAIN

Received: September 3, 2020

Accepted: November 21, 2020

Published: December 15, 2020

Copyright: © 2020 Pierzak et al. This is an open access article distributed under the terms of the Creative Commons Attribution License, which permits unrestricted use, distribution, and reproduction in any medium, provided the original author and source are credited.

Data Availability Statement: All relevant data are within the manuscript and its Supporting Information files.

Funding: European Regional Development Fund in the framework of the Polish Innovative Economy Operational Program (contract no. WNP-POIG.02.02.00-26-023/08) and the Development of Eastern Poland Program (contract no. POPW.01.01.00-26-013/09-04) (Jan Kochanowski University, Kielce, Poland).

Abstract

Total reflection X-ray fluorescence analysis (TXRF) was used to determine chromium, selenium and bromine concentrations in blood serum samples of 50 patients with parenteral nutrition treatment. The concentrations were measured two times, namely in the first day (I measurement) of the treatment and the seventh day (II measurement) after the chromium and selenium supplementation. For comparison purposes also serum samples of 50 patients without nutritional disorders, admitted to a planned surgical procedure to remove the gall bladder (cholecystectomy), were analyzed and treated as the control group. Descriptive statistics of measured concentrations of Cr, Se and Br both for the studied and control groups was determined. In order to check the effectiveness of Cr and Se supplementation, the results of the first and seventh day measurements for studied group were statistically compared with each other, with literature reference values and with the results of the control group (two-group comparison). These comparisons indicate the effectiveness of selenium supplementation in the applied treatment procedure. In the case of Cr and Br concentrations no statistically significant differences were observed. We conclude that monitoring of the concentration of the important trace elements in human serum should be standard procedure in parenteral nutrition treatment. In this monitoring the TXRF technique can be successfully used.

1. Introduction

Total reflection X-ray fluorescence (TXRF) technique [1], thanks to determining the concentrations of many elements simultaneously and the detection limit at the ppb level, has found many interdisciplinary applications [1–3]. One of such applications is the multielemental analysis of medical and biological samples [2–6] and, in particular, human serum samples [7–9].

Competing interests: The authors have declared that no competing interests exist.

Monitoring of blood serum elemental composition is important to the study the influence of environmental pollution, nutrition and/or occupational exposure on human health [10]. Another important aspect of such studies are element concentration changes in serum samples related to various diseases or treatment procedures [9, 11]. The usefulness of the TXRF technique for measuring trace elements in serum samples has already been systematically tested and confirmed [12, 13].

In the presented studies, TXRF technique was used to determine the concentration of elements in blood serum of patients with parenteral nutritional treatment, a difficult therapy requiring supply and substitution of nutrients, taking into account trace elements. It is already known that proper nutrition has important influence on human health by reducing the risk of some diseases and by improving ability to fight off and recovery from illness [14, 15].

Parenteral nutrition, which is intravenous supply of all nutrients, is an alternative body nutrition method in clinical situations with respect to the loss of digestion, absorption, or gastrointestinal motor functions. For this reason, parenteral nutrition was considered one of the most remarkable achievements of the twentieth century medicine [16–19]. In parenteral nutrition, all necessary nutrients are delivered intravenously: proteins, carbohydrates, fats, electrolytes, water and fat soluble vitamins, trace elements and water. Trace elements play an important role in many reactions, on many metabolic pathways that occur in the human body [20]. Therefore, controlled supplementation of trace elements should become an integral element of total parenteral nutrition.

In this work, blood serum samples of 50 patients with parenteral nutrition (studied group from the Clinic of General, Oncological and Endocrinological Surgery, Regional Hospital in Kielce, Poland) were investigated using TXRF technique, in order to measure their elemental composition, mainly to determine chromium and selenium concentrations after supplementation of these elements during parenteral nutrition. In addition to monitoring the effectiveness of selenium and chromium supplementation, the bromine content, element rarely studied especially in conditions of parenteral nutrition and in independence from diet, was also discussed in the study.

Both deficiency and excess of the studied Cr, Se and Br micronutrients have influence on human health and even life. Chromium deficiency in the body disrupts sugar and fat metabolism and damages the circulatory system. Chromium excess damages the circulatory system and causes cancer [20]. Chromium (III) comes in two forms in food products, i.e. the organic and inorganic one. Absorption of the element occurs in the intestines from where it gets into the blood and is distributed to all body tissues. After entering blood, chromium complexes with plasma proteins and is transported to the liver and other organs in these forms.

Selenium deficiency causes cardiomyopathic changes, weakening of the immune system, as well as thyroid dysfunction. Its excess disrupts the metabolism of other trace elements and also damages the parenchymal organs, mainly the liver [20]. Selenium is part of key amino acids, which in turn build enzymes having antioxidant properties. This function has been used to support various types of cancer treatment and neurodegenerative diseases, including Parkinson's disease or Alzheimer's disease. Selenium supplementation is used in Graves' disease and hypothyroidism. The administration of selenium can also strengthen the immune system. It should be remembered that selenium taken in excess is toxic to the body. The direct effect of poisoning with this element is selenosis. In addition, there are studies which showed that selenium consumed in excess may contribute to the developing type II diabetes. For this reason, it is important to check patient plasma selenium level before applying supplementation [21–25].

The physiological role of bromine is still insufficiently known. Bromine is easily absorbed in the digestive tract and enters body fluids. It is usually excreted quickly, but in the absence of iodine, it accumulates in the thyroid gland. In excess, bromine displaces chlorine from

connections and damages nerve cells. While in gaseous form, it is irritating to mucous membranes and causes allergies [20]. Bromine is an essential trace element for assembly of collagen IV scaffolds in tissue development and architecture [26].

The study was performed in two stages: on the first day of parenteral nutritional treatment and seventh day after, and was completed by comprehensive assessment of patient nutritional status. For comparison purposes, also serum samples for the control group (50 persons) were analyzed. The TXRF analysis of the serum elemental composition for both groups was also supported with anthropometric, biochemical and immunological studies (for example: body mass index (BMI), nutritional status, level of the C-reactive protein (CRP), albumin, total protein, leukocytes, lymphocytes), not discuss in presented paper. The aim of our work is not to discuss in detail the role of elements in the human body and the factors that affect their content, but to show that the analysis procedure developed by us using the TXRF method, due to the sensitivity, speed and versatility (multi-elemental analysis), gives the possibility of routine and medical-compliant monitoring of patients undergoing parenteral nutrition.

The presented paper concentrates on statistical analysis and discussion of the result of Cr, Se and Br concentrations determined in serum samples using the TXRF technique. In the paper initial section, the methodological aspects of the measurements are discussed, including: sample collection and preparation, TXRF apparatus calibration procedure and measurement conditions. The statistical analysis used to assess the effectiveness of Cr and Se supplementation and to discuss Br concentration, is focused on the comparison between the experimental results and the reference values, comparison between element concentrations within the studied group (changes between first and seventh day), comparison of the element concentrations between the studied and control groups and on the correlation between elements.

2. Materials and methods

2.1 Sample description

The presented studies included patients of the Clinic of General, Oncological and Endocrinological Surgery, Regional Hospital in Kielce, Poland. The study was approved by the Bioethics Committee at the Jan Kochanowski University in Kielce, Poland (issue 8/2017). The patients signed informed consent to carry out scientific research on the collected biological material.

Nutritional assessment was performed using: a self-study questionnaire containing a nutritional interview, a standardized screening research tool the NRS 2002 (Nutritional Risk Score, NRS 2002), anthropometric, biochemical and immunological tests.

The studied group consisted of patients for whom parenteral nutrition was the only source of nutrients and non-nutrients. There were 50 (100%) persons: 21 (42%) women and 29 (58%) men. Descriptive statistics with respect to age (in years) for patients of the studied group is shown in Table 1. The table presents the mean value, median, standard deviation, minimum and maximum of the age, both for the whole group as well as for men and women separately.

Table 1. Descriptive statistics concerning age (in years) of patients in the studied group.

Parameter	All	Men	Women
**Mean value**	60.8	58.7	63.7
**Median**	65.0	58.0	66.0
**Standard deviation**	14.7	14.6	14.7
**Minimum**	30.0	30.0	30.0
**Maximum**	80.0	80.0	80.0

https://doi.org/10.1371/journal.pone.0243492.t001

Patients included in the study were fed using the *All-in-One* method, involving the use of one food bag. Among nutritional mixture types administered to patients were: Nutriflex Plus, Nutriflex Lipid Peri, Nutriflex Lipid Peri N4, Kabiven Peripheral and Smof Kabiven. Trace elements contained in Addamel supplement and vitamins contained in the product called Cernevit were added to each of the food bags.

Standard chromium supplementation in the form of chromium III hexahydrate in food sacks used in intravenous nutrition in 1 mL of the drug is 5.33 μg/g which corresponds to 0.02 μmol. Standard supplementation of selenium in the form of anhydrous sodium selenite in 1 mL of the drug is 6.90 μg/g, which corresponds to 0.04 μmol. Bromine was not supplemented with nutritional therapy.

All components were dosed in the quantities determined individually, taking into account the clinical condition of the patient, his or her body weight and laboratory test results. Nutrition was carried out via central and peripheral vessels in a continuous infusion, using "MEDINA" feeding pumps for a period not less than 16 hours a day.

The control group consisted of patients without nutritional disorders, admitted to a planned surgical procedure to remove the gall bladder (cholecystectomy). There were 50 (100%) patients: 35 women (70%) and 15 (30%) men. Table 2 presents the descriptive statistics as regards of age (in years) for patients in the control group. As for the studied group, the mean value, median, standard deviation, minimum and maximum of the age, both for whole group and for men and women groups, are presented.

Laboratory analyses of biochemical and immunological parameters were carried out both for the studied and control groups in the Diagnostic Laboratory of the Regional Hospital in Kielce. Venous blood was centrifuged for 15 minutes (4000 rpm). Immediately after centrifugation, blood serum was divided into smaller portions and stored in the laboratory in Ependorf tubes of 1.5 mL volume, at -60˚C. Collected serum samples were systematically forwarded to the Laboratory of X-ray Methods at the Institute of Physics of the Jan Kochanowski University, for analysis of Se, Cr and Br concentrations with TXRF technique.

In the case of studied group, element concentration analysis in serum samples was performed on two occasions: for the samples collected on the first (I measurement) and seventh day (II measurement) of parenteral nutritional treatment. For the patients in the control group, the serum concentration of selenium, chromium and bromine was measured once, before cholecystectomy.

2.2 TXRF technique and measurement conditions

Total reflection X-ray measurements were performed with the S1 Fig (Bruker) [27]. Characteristic X-rays were excited in the samples with the 30 W Mo anode X-ray tube operated at 50 kV with electron current of 0.6 mA. The primary X-ray beam from the tube, monochromated using the Ni/C multilayer monochromator to 17.5 keV, was directed onto the studied sample below the critical angle. Fluorescence X-rays from the samples were detected with the Peltier-

Table 2. Descriptive statistics concerning age (in years) for patients in the control group.

Parameter	All	Men	Women
**Mean value**	53.0	54.1	52.5
**Median**	52.0	60.0	50.0
**Standard deviation**	15.9	15.7	16.2
**Minimum**	30.0	30.0	30.0
**Maximum**	80.0	74.0	80.0

https://doi.org/10.1371/journal.pone.0243492.t002

cooled XFlash silicon drift detector having energy resolution about 160 eV for Mn-Kα line. The measurements were performed in air. Spectrometer software (SPECTRA 7) [28] facilitates both qualitative analysis of the spectrum and the quantitative analysis of the sample elemental composition.

In order to validate the TXRF analysis, the reference control serum solution was investigated on daily basis at the beginning of the studied sample measurement. Measurement time was 1800 s. This reference serum solution contained the following elements with known concentration: V (0.7 mg/L), Co (0.7 mg/L), As (0.7 mg/L), Gd (1.4 mg/L), W (1.4 mg/L) and, as an internal standard, Ga (1.4 mg/L). These elements were added to the human serum because naturally do not occur in the serum (Cr, Br and Se are naturally in the human serum). The set of reference elements covered characteristic radiation both for K and L series, in the energy range from about 4.9 keV to 10.5 keV. This reference serum sample was prepared from monoelemental reference standard solution from Merck KgaA (As, Gd, V) and Ultra Scientific Solution (Ga, Co, W), and human blood serum. Concentration level for all standard solutions was 1000 mg/L ± no more than 6 mg/L. Two-step preparation procedure was as follows: 1) 0.1 mL V (1000 mg/L) + 0.1 mL Co (1000 mg/L) + 0.1 mL As (1000 mg/L) + 0.2 mL Gd (1000 mg/L) + 0.2 mL W (1000 mg/L) + 0.2 mL Ga (1000 mg/L) + 9.1 mL H_2O; 2) 0.7 mL of solution obtained in the first step + 9.3 mL of human blood serum. In Table 3 the results of the N = 20 quality control measurements are presented. There are the mean values, standard deviations, minimum and maximum values of concentration of vanadium (V), cobalt (Co), arsenic (As), gadolinium (Gd) and tungsten (W). In the brackets, also nominal values (in mg/L) are given.

From the table, it can be concluded that experimental mean values of concentration agree with the reference values. The best agreement is observed for Co and Gd (1%), while for the rest of the elements it is on the level 5–9%. Concentration values were stable during control measurements that resulted in relative standard deviation 3–5%. Table 3 also shows detection limit for the reference elements analyzed. Detection limit (C_{DL}) for the TXRF technique is usually calculated using the following formula [29]: $C_{DL} = 3C\sqrt{N_{bckg}}/N$, where C is element concentration, N—peak area of characteristic X-ray line, N_{bckg}—corresponding background area. The value of the detection limit depends on the time of measurements and it is sometimes converted to 1000 s for better comparison. The lowest value of DL, 9 μg/L (ppb), was observed for As. Concerning elements discussed within presented study, namely Cr, Se and Br, the values of the detection limit, calculated from reference serum solution, were on the level: 32 μg/l (ppb) for Cr, and 8 μg/l (ppb) both for Se and Br.

Serum samples of the patients from the studied and control groups were prepared according to the following procedure: 0.8 mL of a sample was mixed with 0.05 mL of Ga (100 μg/g) used as an internal standard. Next, 5 μl of solution was deposited on a quartz sample carrier

Table 3. Concentration of vanadium (V), cobalt (Co), arsenic (As), gadolinium (Gd) and tungsten (W) in serum reference solution.

Element	Mean value mg/L	Standard deviation mg/L	Minimum mg/L	Maximum mg/L	DL mg/L
**V (0.7 mg/L)**	0.666	0.035	0.629	0.758	0.043
**Co (0.7 mg/L)**	0.705	0.028	0.677	0.801	0.019
**As (0.7 mg/L)**	0.760	0.019	0.738	0.826	0.009
**Gd (1.4 mg/L)**	1.38	0.046	1.30	1.53	0.034
**W (1.4 mg/L)**	1.30	0.049	1.26	1.48	0.015

Mean values, standard deviations, minimum (Min) and maximum (Max) concentration values and mean detection limit (DL) are presented for N = 20 quality control measurements. Additionally, nominal values (in mg/L) are given in the brackets.

https://doi.org/10.1371/journal.pone.0243492.t003

and dried on a heating plate at a temperature of 40˚C. The dry residuum was next analyzed using the TXRF technique. The concentrations of the following elements were determined: P, S, Cl, K, Ca, Cr, Mn, Fe, Ni, Cu, Zn, Se, Br, Rb, Sr and Pb. In the TXRF measurement, final uncertainty for concentration determination includes both sample preparation systematic errors (volume determination concerning the solutions used, and the accuracy of determining internal standard concentration) and random errors (counting statistics, generator and X-ray tube stability, and the factors of the calibration curve). The systematic error is approximately 5%. Random errors, mainly due to counting statistics, strongly depended on element concentration in the sample. For concentration levels higher than 0.05 mg/L, the uncertainty is on the level of 5–10%, while for concentration levels less than 0.05 mg/L, the uncertainty can even be up to 50% depending on the atomic number of the element.

In presented studies, the element concentration distributions in a large sample population are discussed and the dispersion of distribution is described by the standard deviation. The value of this parameter, because of properties of element concentration distribution in human sample, is higher than uncertainty of the single measurement.

3. Results and discussion

As mentioned before, these studies and statistical analysis focus on Cr, Se and Br concentration changes as the result of the parenteral nutrition treatment. Chromium and selenium were supplemented during the treatment, whereas bromine naturally occurred in the serum. All statistical calculations presented in this paper, were done in STATISTICA 13. In the future analyzes also the rest of the elements determined in the serum samples will be discussed.

3.1 Experimental results for control group

Table 4 presents descriptive statistics of Cr, Se and Br concentration determined among patients of the control group. In the table the mean values, medians, standard deviations, minimum and maximum values are given. Table also includes confidence intervals for confidence level of 95%.

As can be seen from the Table 4, Cr and Se concentrations in serum are on the similar level, with mean values of 0.054 mg/L and 0.056 mg/L, respectively. Concentration median is however higher for Se. Mean value for Br concentration is much higher (2.30 mg/L). The maximum to minimum concentration ratio is the largest for Cr (about 11), next for Se (about 5) and the lowest for Br (about 4). This is reflected in the relative standard deviation value

Table 4. Descriptive statistics of Cr, Se and Br concentration determined among patients of the control group.

Parameter	Cr	Se	Br
	concentration	concentration	concentration
	mg/L	mg/L	mg/L
**Mean value**	0.054	0.056	2.30
**Median**	0.045	0.055	2.21
**Standard deviation**	0.030	0.015	0.721
**Minimum**	0.014	0.023	1.40
**Maximum**	0.148	0.121	5.28
**Confidence interval (95%)**	0.046	0.051	2.10
	0.063	0.060	2.51

Mean values, medians, standard deviations, minimum and maximum values are given. Table also includes confidence intervals for confidence level of 95%.

https://doi.org/10.1371/journal.pone.0243492.t004

Table 5. Descriptive statistics of Cr, Se and Br concentration determined among patients of the studied group, in the first (I measurement) and in the seventh day (II measurement) of treatment.

Parameter	Cr concentration (mg/L)		Se concentration (mg/L)		Br concentration (mg/L)	
**Measurement**	I	II	I	II	I	II
**Mean value**	0.057	0.050	0.045	0.052	1.20	1.21
**Median**	0.048	0.045	0.044	0.030	1.06	1.14
**Standard deviation**	0.030	0.024	0.019	0.021	0.639	0.570
**Minimum**	0.017	0.012	0.011	0.018	0.312	0.332
**Maximum**	0.129	0.111	0.106	0.110	2.94	2.63
**Confidence interval (95%)**	0.048	0.043	0.040	0.046	1.02	1.05
	0.065	0.057	0.051	0.058	1.38	1.37

Mean values, medians, standard deviations, minimum and maximum values are given. Table also includes confidence intervals for confidence level of 95%.

https://doi.org/10.1371/journal.pone.0243492.t005

(standard deviation to mean value ratio, percentage), which for Cr is about 60% while for Se and Br is about 30%.

3.2 Experimental results for studied group

Table 5 presents descriptive statistics of Cr, Se and Br concentration determined among patients of the studied group, both for first and second measurements. Table shows the mean values, medians, standard deviations, minimum and maximum values. The table also includes confidence intervals for confidence level of 95%.

Table 5 illustrates Cr and Se concentrations in serum at a comparable level, similar like for the control group, with mean values in I measurement of 0.057 mg/L and 0.045 mg/L, respectively for Cr and Se, and 0.050 mg/L (Cr) and 0.052 mg/L (Se) in II measurement. Medians are almost equal but for Se in second measurement the median is lower of about 30%. The mean value for Br concentration is higher (1.2 mg/L), but about two times lower comparing with the control group (Table 4). In the first measurement, the maximum to minimum concentration values ratio is the largest for Se (about 10), next for Br (about 9) and for Cr (about 8) while in the second measurement this ratio decreases for Se (6) and Br (8). This is reflected in the changes of the relative standard deviation which for Cr changes from 53% to 48%, for Se from 42% to 40% and for Br from 53% to 47%. Supplementation reduces group heterogeneity in the aspect of the studied element concentrations.

3.3 Comparison of the experimental results with reference values

The experimental values of Cr, Se and Br concentration were compared with concentration reference values for these elements in human serum. The reference values of Cr, Se and Br concentration in human serum, which were determined in our previous studies [8], are presented in Table 6. These results were obtained on the based of analysis of serum samples of 105 normal presumably healthy volunteers from Świętokrzyskie Province (Poland).

In order to compare experimental concentration mean values with the reference values, the statistical one-sample *t* test was applied. In this test a null hypothesis H_0 was as follows: mean

Table 6. Reference values of Cr, Se and Br concentrations in human serum [8].

Element	Reference values (mg/L)
**Cr**	from 0.117 to 0.171
**Se**	from 0.051 to 0.071
**Br**	from 1.58 to 2.67

https://doi.org/10.1371/journal.pone.0243492.t006

values X_1 of element concentration in the group (control or studied (I and II measurement)) is equal to the theoretical (reference) value X_{ref} ($X_1 = X_{ref}$), while an alternative hypothesis H_1 was: mean values are different ($X_1 \neq X_{ref}$). The assumption of this one-sample *t* test requires that studied variable, in this case element concentration, is described by normal distribution. In our earlier studies we presented, however, that element concentration distributions in biomedical samples, are described by log-normal, or more general by log-stable, distribution [30]. The main property of the log-normal distribution is fact that after logarithmic transformation, the set of data being normally distributed is obtained. In presented studies, for Cr, Se and Br concentration distributions (both for I and II measurements) the goodness-of-fit tests were performed, showing that these distribution are not drawn from normal distribution (for significance level 0.05). As expected [30], the assumption about the normality of concentration distribution was fulfilled after data logarithmic transformation. Consequently, statistical verifications were carried out for transformed set of data. Additionally, at the initial stage of statistical analysis the gender of the patient was not taken into account.

The interpretation of the statistical tests, performed for significance level $\alpha = 0.05$, was as follows: in the control group Cr concentration is lower than the reference value (p value < 0.05), while Se and Br concentrations are equal to the reference values.

Analyzing the results for the studied group has showed that in the first measurement (I), in the first day of supplementation, concentration mean values of all discussed elements (Cr, Se and Br) are lower than reference values (p value < 0.05). In the case of the second (II) serum analysis, in the seventh day of supplementation, mean values of Cr and Br concentration are lower than reference values (p value < 0.05). Significant change was observed for Se concentration, because in the second measurement selenium concentration was equal to the reference value (p value $= 0.26 > 0.05$). Concluding in one-sample test the statistically significant effectiveness of Se supplementation was evidenced.

3.4 Comparison of the element concentrations in studied group

Comparison of the element concentrations in studied group was performed using paired-sample *t* test. In this test a null hypothesis H_0 was as follows: mean values of element concentration in the first day (measurement I) and in the seventh day (measurement II) of parenteral nutritional are equal ($X_1 = X_2$), while an alternative hypothesis H_1 was: mean values are different ($X_1 \neq X_2$). The assumption about the concentration distribution was fulfilled after logarithmic transformation of the data. Additionally, at the initial stage of statistical analysis the gender of the patient was not taken into account. The results of the statistical comparison are presented in Table 7. The p values are compared with significance level α assumed as 0.05 ($\alpha = 0.05$).

Statistical comparison showed that the statistically significant differences in the mean values of element concentrations between the first and the seventh day of parenteral nutrition were observed for selenium (p value much lower than assumed significant level 0.05).

Table 7. Comparison results of element concentrations for studied group in the first day (measurement I) and in the seventh day (measurement II) of parenteral nutrition. The analysis was performed on logarithmically transformed data.

Variable	ln(X) of Cr		ln(X) of Se		ln(X) of Br	
**Measurement**	I	II	I	II	I	II
**Mean value**	-3.00	-3.13	-3.19	-3.04	0.037	0.082
**Sample number**	50	50	50	50	50	50
**p value**	0.166 ($p > \alpha$)		**0.006** ($p < \alpha$)		0.420 ($p > \alpha$)	

The p values were compared with significance level α assumed as 0.05 ($\alpha = 0.05$).

https://doi.org/10.1371/journal.pone.0243492.t007

In further statistical comparison of element concentrations in the studied group, patient gender was taken into account. Finally, no statistically significant differences between element concentration in the first day of parenteral nutrition were observed, while statistically significant differences were obtained in Se concentration in the seventh day of parenteral nutrition. Additionally, it can be concluded that Se supplementation is slightly more effective for women.

3.5 Comparison of the element concentrations between studied and control groups

Comparison of element concentrations between the studied and control groups was performed using a two-sample *t* test. In this test a null hypothesis H_0 was as follows: mean values of element concentration in the first day (the seventh day) of parenteral nutrition and in the control group are equal ($X_1 = X_2$), while an alternative hypothesis H_1 was: mean values are different ($X_1 \neq X_2$). The assumption about concentration distribution was fulfilled after logarithmic data transformation. Additionally, at the initial stage of statistical analysis the gender of the patient was not taken into account. The significance level α was assumed as 0.05 ($\alpha = 0.05$).

Comparison results of element concentrations between the studied and control groups can be summarized as follows: 1) in the case of Cr, there are not statistically significant differences between concentration in the first day (and seventh day) and the control group, 2) in the case of Se, there are statistically significant differences between concentration in the first day and the control group, after supplementation, however, concentration levels are equal, 3) in the case of Br, there are statistically significant differences between concentration in the first day (and seventh day) and the control group.

Graphic presentation of comparison concerning Se concentrations in the control and studied (I and II measurement) groups is shown in the Fig 1. In the figure, raw data are presented as well as mean values and standard deviations of concentration for each group. Figure also presents range of reference values for Se concentration in human serum [8]. The data are presented after logarithmic transformation which also was used in one- and two-sample tests. It can be observed that for I measurement in the studied group, the mean value of Se concentration is lower comparing with II measurement as well as with the control group (statistically significance changes). The p value ($p = 0.006$) is related to comparison of Se concentration in the studied group for the I and the II measurements. Additional observation is that the studied group is characterized by a higher value of the standard deviation of Se concentration.

3.6 Correlation between elements

Possible correlations between the elements were calculated for logarithmically transformed element concentration, using Pearson's correlation coefficient. Statistical significance correlations were confirmed by test checking that correlation coefficient was statistically different from zero. In the control group, for all patients, it was observed for Se and Br (Pearson's correlation coefficient = 0.429). Additionally, the same conclusions for Se and Br were observed taking into account the patient gender, obtaining similar values of Pearson's correlation coefficient, respectively, 0.421 for women and 0.561 for men. The interpretation of this test as follows: higher (lower) Se concentration in serum is accompanied by higher (lower) concentration of Br.

In the case of the studied group, due to the double measurement of element concentrations in serum sample, a new variable containing result both of the first and the second measurements was defined in order to determine element correlations, namely: the difference of concentration logarithms. A variable defined in this manner is described by normal distribution which is the assumption for the Pearson's correlation coefficient. As a result, the statistically

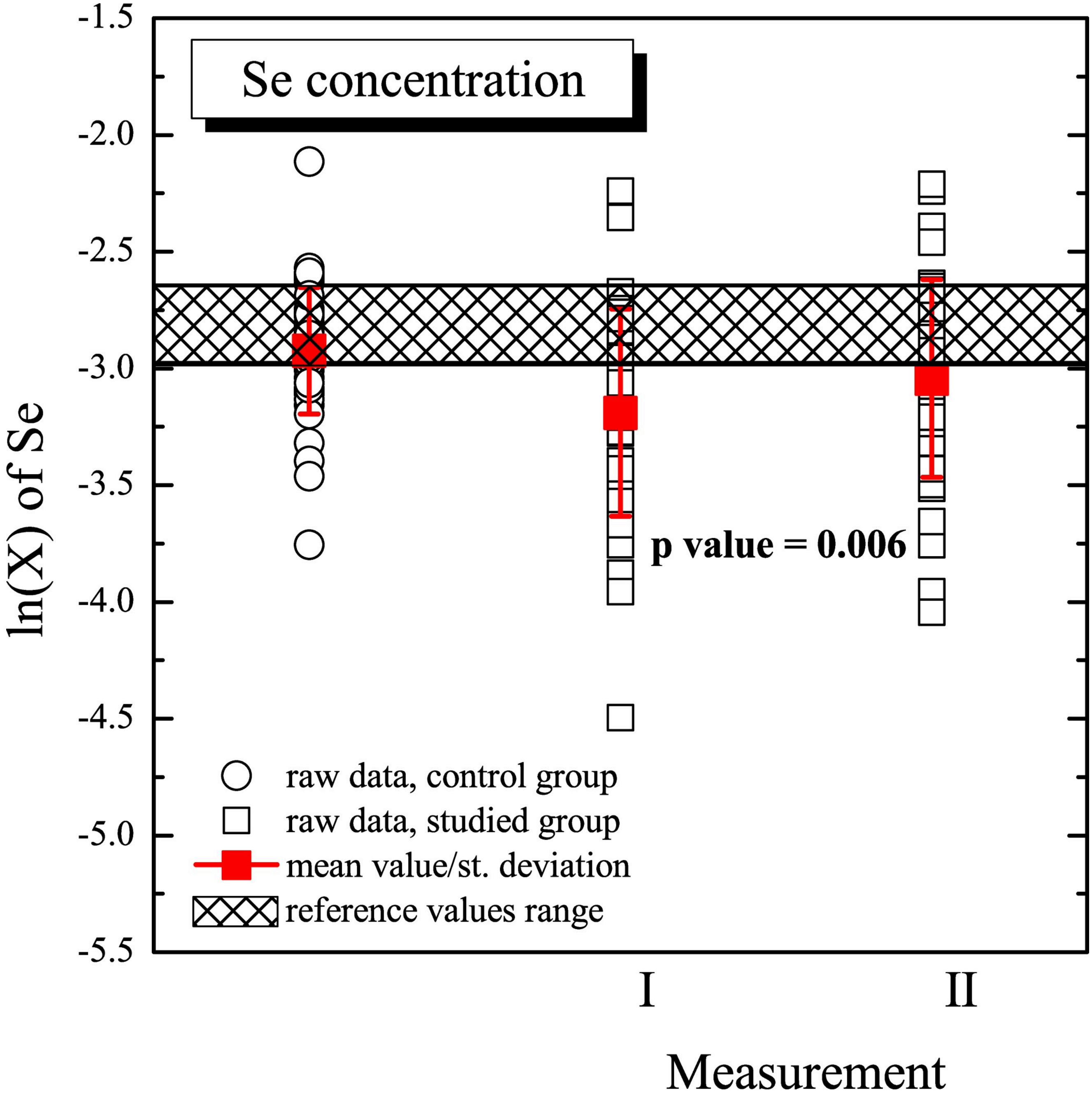

Fig 1. Graphic presentation of comparison of Se concentrations in the control and studied (I and II measurement) groups. In the figure, the raw data are presented as well as the mean values and standard deviations of concentration for each group and reference values range of Se concentration in human serum. The p value (p = 0.006) was related to the comparison of Se concentration in the studied group for the I and the II measurements. The data are presented after logarithmic transformation.

https://doi.org/10.1371/journal.pone.0243492.g001

significant difference was found between Se and Br concentration changes (0.323, all patients), Cr and Br concentration changes (0.538, women group), Se and Br concentration changes (0.438, women group). In the case of men, a relatively high value of Pearson's coefficient was obtained for Se and Br concentration change comparison (0.310) but this correlation was not confirmed statistically.

In correlation also age of the patients was taken into account but both for the control group and the studied group (all patients, women group, men group) no correlations between Cr, Se and Br concentrations and age of the patients were observed.

Supporting information

S1 Fig. Spectrum of the characteristic radiation emitted from the serum sample, obtained using total reflection X-ray fluorescence analysis (S1 Fig). In the spectrum Cr, Se and Br K-α lines are observed. The intensity of the line is converted to element concentration.
(PDF)

Author Contributions

Conceptualization: Monika Pierzak, Stanisław Głuszek.

Data curation: Monika Pierzak.

Formal analysis: Monika Pierzak, Aldona Kubala-Kukuś, Stanisław Głuszek.

Funding acquisition: Monika Pierzak, Dariusz Banaś, Stanisław Głuszek.

Investigation: Monika Pierzak, Ilona Stabrawa, Jolanta Wudarczyk-Moćko.

Methodology: Monika Pierzak.

Project administration: Monika Pierzak, Dariusz Banaś.

Resources: Monika Pierzak, Aldona Kubala-Kukuś, Dariusz Banaś, Stanisław Głuszek.

Supervision: Monika Pierzak.

Validation: Monika Pierzak, Aldona Kubala-Kukuś, Jolanta Wudarczyk-Moćko.

Visualization: Monika Pierzak, Aldona Kubala-Kukuś.

Writing – original draft: Aldona Kubala-Kukuś, Dariusz Banaś.

Writing – review & editing: Monika Pierzak, Aldona Kubala-Kukuś, Dariusz Banaś.

References

1. Klockenkämper R, von Bohlen A. Total Reflection X-Ray Fluorescence Analysis and Related Methods. Wiley: New Jersey; 2015.
2. Liui Y. Low power total reflection x-ray fluorescence spectrometry: A review. Adv. X-Ray. Chem. Anal., Japan 2015; 46: 13–26.
3. Salomone VN, Riera M, Muniain C. Total reflection x-ray fluorescence: A valuable analytic tool for environmental and biological studies. Int. J. Exp. Spectroscopic Tech. 2017; 2:011: 1–6.
4. Magalhães T, Carvalho M, von Bohlen A, Becker M. Study on trace elements behaviour in cancerous and healthy tissues of colon, breast and stomach: Total reflection X-ray fluorescence applications. Spectrochim. Acta B. 2010; 65: 493–498.
5. Kubala-Kukuś A, Arabski M, Stabrawa I, Banaś D, Różański W, Lipiński M, et al. Application of TXRF and XRPD techniques for analysis of elemental and chemical composition of human kidney stones. X-Ray Spectrom. 2017; 46: 412–420.
6. Majewska U, Łyżwa P, Kubala-Kukuś A, Banaś D, Wudarczyk-Moćko J, Stabrawa I, et al. Total reflection x-ray fluorescence in medical applications: elemental analysis of human urine. Spectrochim. Acta B. 2018; 147: 121–131.
7. Majewska U, Banaś D, Braziewicz J, Kubala-Kukuś A, Pajek M, Sychowska I, et al. X-ray fluorescence techniques in medical applications: reference values of elements in human serum, urine and hair. Acta Phys. Pol. A. 2014; 125: 864–868.

8. Majewska U, Łyżwa P, Łyżwa K, Banaś D, Kubala-Kukuś A, Wudarczyk-Moćko J, et al. Determination of element levels in human serum: Total reflection x-ray fluorescence applications. Spectrochim. Acta B. 2016; 122: 56–61.
9. Kubala-Kukuś A, Banaś D, Braziewicz J, Majewska U, Pajek M, Wudarczyk-Moćko J, et al. Analysis of copper concentration in human serum by application total reflection x-ray fluorescence method. Biol. Trace Elem. Res. 2014; 158: 22–28. https://doi.org/10.1007/s12011-013-9884-4 PMID: 24573405
10. Stosnach H, Mages M. Analysis of nutrition-relevant trace elements in human blood and serum by means of total reflection X-ray fluorescence (TXRF) spectroscopy. Spectrochim. Acta B. 2009; 64: 354–356.
11. Ashraf A, Stosnach H, Parkes HG, Hye A, Powell J. Pattern of altered plasma elemental phosphorus, calcium, zinc, and iron in alzheimers disease. Scientific Reports. 2019; 9: 3147. https://doi.org/10.1038/s41598-018-37431-8 PMID: 30816126
12. Zarkadas C, Karydas A, Paradellis T. Applicability of direct total reflection x-ray fluorescence analysis in the case of human blood serum samples. Spectrochim. Acta B. 2001; 56: 2219–2228.
13. Jeffery J, Frank AR, Hockridge S, Stosnach H, Costelloe SJ. Method for measurement of serum copper, zinc and selenium using total reflection x-ray fluorescence spectroscopy on the picofox analyser: Validation and comparison with atomic absorption spectroscopy and inductively coupled plasma mass spectrometry. Ann. Clin. Biochem. 2018; 56: 170–178. https://doi.org/10.1177/0004563218793163 PMID: 30056759
14. Wrzochal A, Gładyś-Jakubczyk A, Suliga E. Evaluation of diet in preschool-age children with down syndrome—preliminary examination. Medical Studies. 2019; 35: 128–138.
15. Solecka M, Głuszek S. Nutritional intervention during radiotherapy of head and neck cancers. Medical Studies. 2018; 34: 153–159.
16. Goździalska A, Jaśkiewicz J, (eds.). Współczesne kierunki w medycynie prewencyjnej. Krakowskie Towarzystwo Edukacyjne sp. z o.o.—Oficyna Wydawnicza AFM. Kraków; 2013.
17. Pertkiewicz M, Korta T, (eds.). Standardy żywienia pozajelitowego i żywienia dojelitowego. Wydawnictwo Lekarskie PZWL. Warszawa; 2005.
18. Spodaryk M, Paluszkiewicz P. Praktyczne aspekty leczenia żywieniowego. Wydawnictwo Uniwersytetu Jagiellońskiego. Kraków; 2008.
19. Lee H, Raman GV. A Handbook of Parenteral Nutrition. Springer. Science. Buisness Media. B. V.; 1990.
20. Kabata-Pendias A, Mukherjee A. Trace Elements from Soil to Human. Springer; 2007.
21. Klecha B, Bukowska B. Selen w organizmie człowieka—charakterystyka pierwiastka i potencjalne zastosowanie terapeutyczne. Bromat. Chem. Toksykol. 2016; 4: 818–829.
22. Duntas L, Benvega S. Selenium: an element for life. Endocrine. 2014; 48: 756–775. https://doi.org/10.1007/s12020-014-0477-6 PMID: 25519493
23. Fairweather-Tait SJ, Bao Y, Broadley M, Collings R, Ford D, Hesketh J, et al. Selenium in human health and disease. Antioxid. Redox Signal. 2011; 14: 1337–1383. https://doi.org/10.1089/ars.2010.3275 PMID: 20812787
24. Fernandes A, Gandin V. Selenium compounds as therapeutic agents in cancer. Biochim. Biophys. Acta. 2015; 1850: 1642–1660. https://doi.org/10.1016/j.bbagen.2014.10.008 PMID: 25459512
25. Steinbrenner H, Al-Quraishy S, Dkhil M, Wunderlich F, Sies H. Dietary selenium in adjuvant therapy of viral and bacterial infections. Adv. Nutr. 2015; 6: 73–82. https://doi.org/10.3945/an.114.007575 PMID: 25593145
26. McCall AS, Cummings CF, Bhave G, Vanacore R, Page-McCaw A, Hudson BG. Bromine is an essential trace element for assembly of collagen IV scaffolds in tissue development and architecture. Cell. 2014; 157: 1380–1392. https://doi.org/10.1016/j.cell.2014.05.009 PMID: 24906154
27. User Manual S2 PICOFOX. TXRF Spectrometer for element analysis. Bruker NanoGmbH, Berlin; 2012.
28. User Manual SPECTRA 7. Software for acquisition and evaluation of XRF spectra. Bruker NanoGmbH, Berlin; 2011.
29. Klockenkämper R. Total Reflection X-Ray Fluorescence Analysis. Willey. New York; 1997.
30. Kubala-Kukuś A, Braziewicz J, Pajek M. Total-reflection x-ray fluorescence studies of trace elements in biomedical samples. Spectrochim. Acta B. 2004; 59: 1283–1289.
